# Discovery of the Linear Region of Near Infrared Diffuse Reflectance Spectra Using the Kubelka-Munk Theory

**DOI:** 10.3389/fchem.2018.00154

**Published:** 2018-05-07

**Authors:** Shengyun Dai, Xiaoning Pan, Lijuan Ma, Xingguo Huang, Chenzhao Du, Yanjiang Qiao, Zhisheng Wu

**Affiliations:** Key Laboratory of TCM-Information Engineering of State Administration of TCM, Pharmaceutical Engineering and New Drug Development of Traditional Chinese, Medicine of Ministry of Education, Beijing University of Chinese Medicine, Beijing, China

**Keywords:** Kubelka-Munk theory, Near infrared (NIR) diffuse reflectance spectroscopy, particle size, PLS, harpagoside, *Radix Scrophulariae*

## Abstract

Particle size is of great importance for the quantitative model of the NIR diffuse reflectance. In this paper, the effect of sample particle size on the measurement of harpagoside in *Radix Scrophulariae* powder by near infrared diffuse (NIR) reflectance spectroscopy was explored. High-performance liquid chromatography (HPLC) was employed as a reference method to construct the quantitative particle size model. Several spectral preprocessing methods were compared, and particle size models obtained by different preprocessing methods for establishing the partial least-squares (PLS) models of harpagoside. Data showed that the particle size distribution of 125–150 μm for *Radix Scrophulariae* exhibited the best prediction ability with ${\text{R}}_{\text{pre}}^{2}$ = 0.9513, RMSEP = 0.1029 mg·g^−1^, and RPD = 4.78. For the hybrid granularity calibration model, the particle size distribution of 90–180 μm exhibited the best prediction ability with ${\text{R}}_{\text{pre}}^{2}$ = 0.8919, RMSEP = 0.1632 mg·g^−1^, and RPD = 3.09. Furthermore, the Kubelka-Munk theory was used to relate the absorption coefficient *k* (concentration-dependent) and scatter coefficient *s* (particle size-dependent). The scatter coefficient *s* was calculated based on the Kubelka-Munk theory to study the changes of *s* after being mathematically preprocessed. A linear relationship was observed between *k*/*s* and absorption *A* within a certain range and the value for *k*/*s* was >4. According to this relationship, the model was more accurately constructed with the particle size distribution of 90–180 μm when *s* was kept constant or in a small linear region. This region provided a good reference for the linear modeling of diffuse reflectance spectroscopy. To establish a diffuse reflectance NIR model, further accurate assessment should be obtained in advance for a precise linear model.

## Introduction

The implementation of process analytical technology (PAT) in the pharmaceutical industry is intended to enhance the quality of products through the measurement of critical quality and performance parameters (Roggo and Ulmschneider, 2008). Near infrared spectroscopy (NIRS) is regarded as a vital tool for the implementation of PAT, as it is increasingly used in pharmaceutical research and development due to its high analysis speed, low-cost, and non-destructive characteristics (De Beer et al., 2011). NIR spectra of chemical species (consisting of C–H, N–H, O–H, and S–H bonds; Sarraguça et al., 2011) can be used to predict their chemical and physical properties (Prieto et al., 2009).

The NIR technology includes two main parts that are transmission spectroscopy and diffuse reflectance spectroscopy. The selection of spectral form is mainly based on the state of samples (i.e., transmission spectroscopy is suitable for liquid samples such as herbal extracts and liquid preparations, while diffuse reflectance spectroscopy is generally used for solid samples such as pharmaceutical powders or granules). Diffuse reflectance spectroscopy is an analytical technique that measures the diffuse reflection of different wavelengths of light to obtain the surface information of the materials.

Various physical, chemical, and biochemical properties in Mediterranean soils were NIR predicted (Zornoza et al., 2008). Chen et al. employed an NIR model for the analysis of total polyphenol content in green tea (Chen Q. et al., 2008). Classification accuracy of about 100 % was obtained by discriminant and classification tree analyses of 82 honey samples by diffuse reflectance mid-infrared Fourier transform spectroscopy (DRIFTS) (Bertelli et al., 2007). Borin et al. utilized NIR technology for the simultaneous quantification of some common adulterants (starch, whey, or sucrose) found in milk powder samples (Borin et al., 2006). All these investigations have illustrated the trend of using NIR technology to predict physical and chemical information.

Recently, the application of NIR in studying Chinese herbal medicine (CHM) has dramatically increased such as discrimination analysis and quality control for various samples e.g., raw materials, excipients, and dosage forms. Wu et al. used the NIR and different PLS models to quantify the baicalin contents of Yinhuang oral solution based on a total error concept (Wu et al., 2013). Chen et al. employed NIR to distinguish Ganoderma lucidum samples collected from different geographical origins using principal component analysis (PCA) and discriminant analysis algorithms (Chen Y. et al., 2008).

On the other hand, it is well known that the particle size of sample affects NIR spectra. Several studies have been published on the effect of particle size on the determination of drug content in mixed powder products (Norris and Williams, 1984; Aucott and Garthwaite, 1988; Bull, 1991). Franke et al. (1998) reported the particle size determination of lactose using chemometrics-based NIR spectra. However, they did not mention any basic principle to determine particle size in the experiments. Paskatan et al. (2001) reviewed theoretical and practical particle size analysis of powder by NIR spectroscopy. But they did not show the relationship between the basic light scattering principle and the particle size of main contents.

Kubelka-Munk theory (Otsuka, 2004) is the basic quantitative theory of NIRS. The particle size of sample affects the light scattering, directly influencing model construction. It was shown that an accurate knowledge of the particles is crucial in the product development (Blanco and Peguero, 2008). Meanwhile, the differences in CHM particle size could result in different optical path lengths and multiplicative light scattering effects (Jin et al., 2012). Thus, it is important to establish an expeditious method to determine the particle size of CHM.

However, there were a few NIR studies on the simultaneous determination of particle size and active pharmaceutical ingredients of CHM. Wu Z. S. et al. demonstrated that the particle size affected NIR measurement of saikosaponin A in *Bupleurum chinense DC* (Wu et al., 2015). Bittner et al. employed a successful application of NIR spectroscopy in combination with multivariate data analysis (MVA) for the simultaneous identification and particle size determination of amoxicillin trihydrate particles (Bittner et al., 2011).

*Scrophularia* radix (Xuanshen), the root of *Scrophularia ningpoensis Hemsl*., was a typical CHM with a history going back over 1000 years (The State Pharmacopoeia Commission of People's Republic of China, 2015). It is originally from Zhejiang province and it is a component of the natural herbal supplement named “Zhe Ba Wei.” The major ingredients of *Scrophularia* radix are iridoids, and harpagoside is one of the main bioactive components with antioxidant, antimicrobial and antitumor activities (Miyazawa and Okuno, 2003; Jing et al., 2011).

In this study, *Scrophularia* radix was taken as an example and harpagoside was regarded as an API of *Scrophularia* radix. HPLC was used as a reference method to determine the harpagoside content. NIR was used to monitor the prediction potential of the models of single particle size and mix particle size simultaneously. To our best knowledge, this paper is the first to study on particle size and harpagoside determination in *Scrophularia* radix with NIR diffuse reflectance spectroscopy. The differences between single particle size model and mix particle size model from the perspective of the Kubelka-Munk theory were explained.

## Materials and method

### Materials

Ten batches of *S. ningpoensis* Hemsl. radix were gifted from Daozhen (Guizhou, China), three representative samples were taken from each batch. All samples were identified by Prof. Chunsheng Liu (Beijing University of Chinese Medicine, China). Harpagoside reference standard (lot: 111730-201307) was purchased from the National Institutes for Food and Drug Control (Beijing, China). Acetonitrile (Fisher Scientific, Pittsburgh, PA) was of HPLC-grade. Acetic acid (Beijing Chemical Works, Beijing, China) was of analytical grade. Deionised water was purchased from Hangzhou Wahaha Co., Ltd (Zhejiang, China).

### Preparation of samples

*Scrophularia* radix samples were crushed into pieces by a disintegrator after brushing off soil dust from the surface. Thirty samples of *Scrophularia* radix were then pulverized with a blender and screened through a 10-mesh sieve. Finally, the powders were divided into four parts. One part was used for HPLC determination of the harpagoside content. The remaining parts were then smashed and screened through 24-, 50-, 65-, 80-, 100-, 120-, and 150-mesh sieves.

An amount of each sieved sample of *Scrophularia* radix powder (1 g) was accurately weighed and placed in a 100-mL Erlenmeyer flask. The sample was extracted with 50 mL of 50% ethanol under ultrasonic vibration (40 kHZ, 220 V) for 45 min. After cooling to room temperature, the solution was filtered through a 0.45-μm membrane filter for HPLC analysis.

### NIR equipment and measurement

The NIR spectra were recorded by a XDS Rapid Content Analyser and VISION software (Metrohm NIR Systems, Florida, USA). The wavelength range for the spectra was 780–2,500 nm. Each spectrum was an average of 64 scans with air as the background, and the wavelength increment was of 0.5 nm. Unless stated otherwise, each sample was measured in triplicate and its mean value was used in the subsequent analysis.

### HPLC method

A certain amount of harpagoside standard was accurately weighed with an XS205DU electronic balance (Mettler Toledo, Greifensee, Switzerland) and then dissolved in 100 mL of methanol to obtain the concentration of 0.02432 mg·mL^−1^.

HPLC analysis of *Scrophularia* radix (according to Chinese Pharmacopoeia, 2010 ed) was carried out using a Waters 2695 HPLC system, Waters 2996 DAD detector and auto-sampler (Waters Technologies, Palo Alto, CA). Ten microliters aliquots of the sample solutions were chromatographically analyzed in gradient elution mode on an octadecylsilyl column [250 × 4.6 mm, 5 μm (Dikma, China)] with the mobile phase consisting of acetonitrile and 0.4% acetic acid (*v*/*v*) at a flow rate of 1.0 mL·min^−1^ (Table 1). The column temperature was kept at 30°C and the detection wavelength set at 280 nm. This chromatographic method exhibited good linearity (Y = 3 × 10^6^X−104747, *R*^2^ = 0.9998) over the concentration range 0.04864–0.02432 mg·mL^−1^.

**Table 1:** HPLC gradient elution of *Scrophularia* radix extract.

**Time/min**	**A/%**	**B/%**
0–10	5–10	95-90
10–25	10–33	90–67
25–35	33–50	67–50
35–40	50–60	50–40
40–45	60–70	40–30
45–55	70–80	30–20
55–60	80–5	20–95

### Software

Data analysis was performed by the Unscrambler version 9.6 software package (CAMO Software AS, Oslo, Norway) and home-made routines programmed in MATLAB code (MATLAB v7.0, Math Works, Natick, MA). Following the Kennard-Stone algorithm, 210 samples were divided into 140 calibration samples and 70 validation samples. The root mean square error of calibration (RMSEC), root mean square error of cross-validation (RMSECV), root mean square error of prediction (RMSEP) and corresponding *R*^*2*^ were used to evaluate the PLS model.

In order to establish a robust harpagoside model, a number of preprocessing methods were selected. For instance, multiplicative scatter correction (MSC) and standard normal variate (SNV) were used to eliminate redundant effects of particle size. Derivative methods including first derivative (1D) and second derivative (2D) were obtained to reduce baseline variations observed in original diffuse reflectance spectra and to enhance spectral features. Meanwhile, a nine-point Savitzky-Golay smoothing filter (SG) was employed to depress the background noise amplified by the derivative. For the particle size model, MSC, SNV, and second derivative were not appropriate for an effect to be modeled, so 1D + SG, normalization and baseline subtraction were used. Leave-one-out cross-validation was used to validate the validity of methods. The lowest predicted residual sum of squares (PRESS) value was used to determine the optimum latent variables.

### Quantitative models of NIR diffuse reflectance using the Kubelka-Munk theory

Kubelka-Munk theory is the theoretical basis for the establishment of quantitative models of NIR diffuse reflectance and its function is as follows (Otsuka, 2004):

$$\begin{array}{l} \text{f}\left(R_{\infty }\right)=\frac{{\left(1-R_{\infty }\right)}^{2}}{2}R_{\infty }=\frac{k}{s} \end{array}$$

According to the Kubelka-Munk function, reflectance is inversely to proportional to the light-scattering coefficient (*s*), and the *s* value is inversely proportional to particle size.

The absorbance of NIR diffuse reflectance is expressed by the Kubelka-Munk equation:

$$\begin{array}{l} \text{A}=-\text{lg}\left[1+\frac{k}{s}-\sqrt{{\left(\frac{k}{s}\right)}^{2}+2\left(\frac{k}{s}\right)}\right] \end{array}$$

## Results and discussion

### Spectral characteristics of NIR diffuse reflectance spectra of different particle size samples

**Figure 1:**
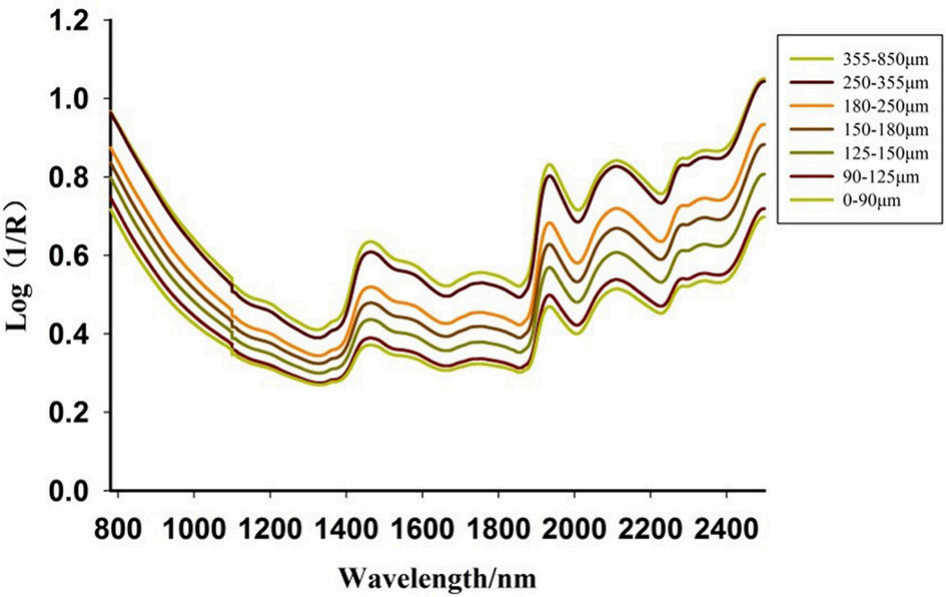
NIR diffuse reflectance spectra of Scrophularia radix.

The representative raw spectra of *Scrophularia* radix with different particle sizes are shown in Figure 1 i.e., the spectral profiles were similar in shape. However, the main influences of particle size variation on diffuse reflectance spectra was the baseline offset. The well-known phenomenon that larger particles showed a stronger absorption, illustrates that the particle size is vital to the response. Some weak absorption peaks were demonstrated in the second overtone region (SCOT, 1,000–1,400 nm) of the fundamental C-H stretching bands, while much fluctuations in the region of first combination-overtone (FCOT, 1,400–2,040 cm^−1^) and combination region (CR, 2,040-2,500 nm) were observed. Those absorption peaks might be caused by the diffuse reflectance on different particle sizes.

### HPLC determination of harpagoside content in *Scrophularia* radix

**Figure 2:**
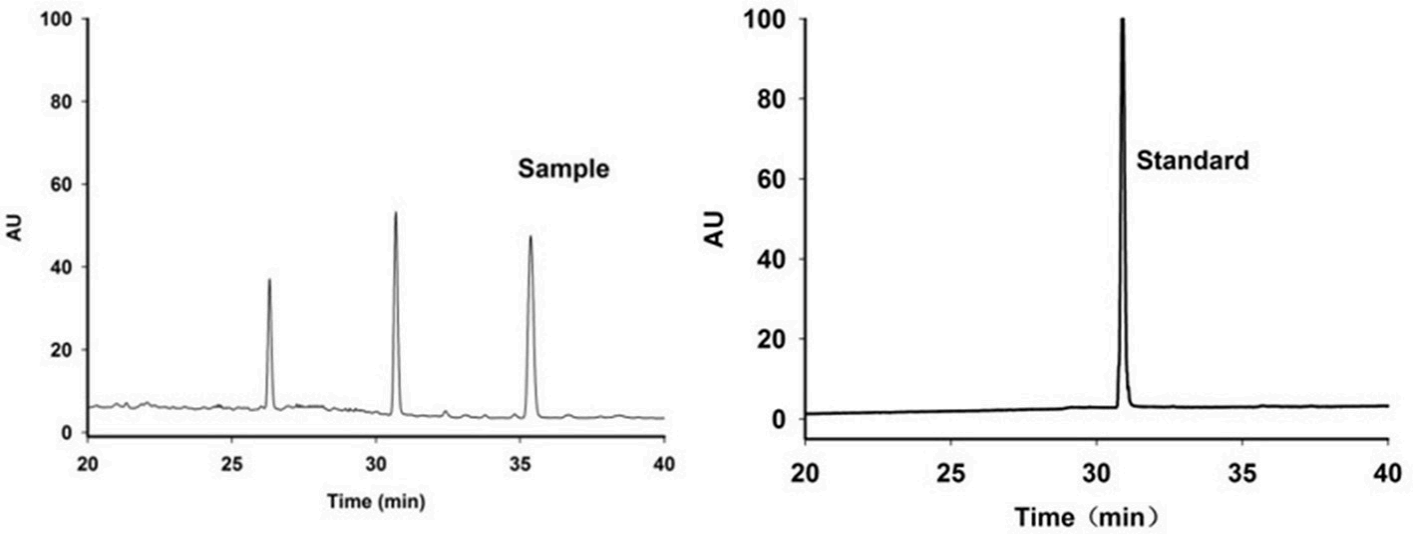
Representative HPLC chromatograms of Scrophularia radix sample and harpagoside standard.

**Figure 3:**
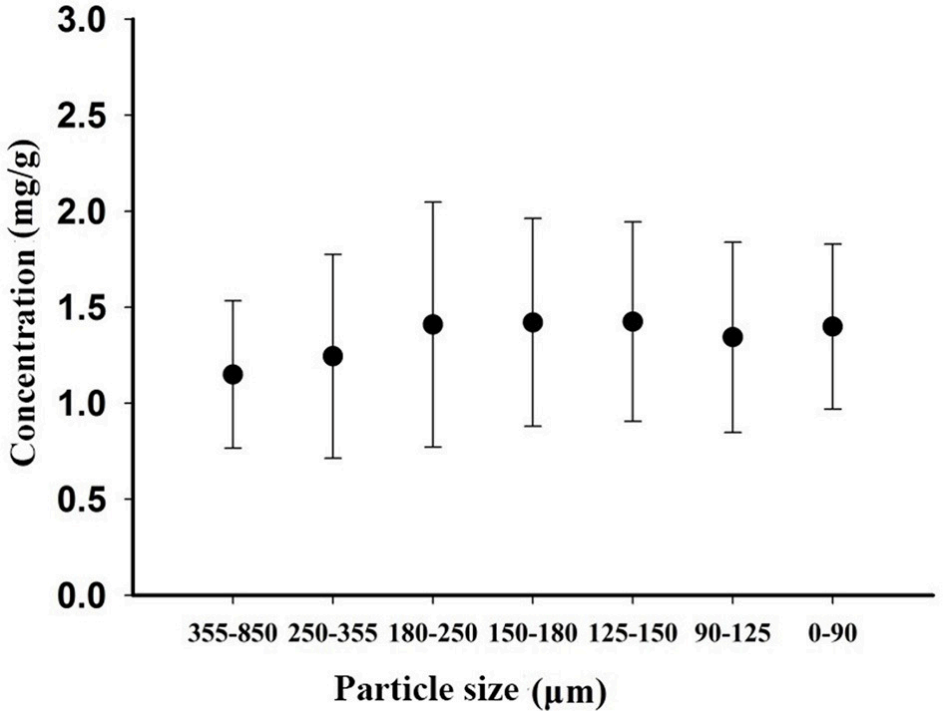
Harpagoside concentration of 30 samples of different particle sizes.

The HPLC chromatograms of the representative sample and standard are shown in Figure 2. The retention time of harpagoside in a sample extract was the same as that for the standard solution. Figure 3 shows the harpagoside concentration of 30 samples. There is a significant difference in harpagoside concentration of samples of different particle sizes. The biggest difference of the particle sizes was located in the range of 180–250 μm, but the overall concentration design was suitable for the modeling.

### PLS models for NIR diffuse reflectance data using *Scrophularia* radix of each single particle size

**Figure 4:**
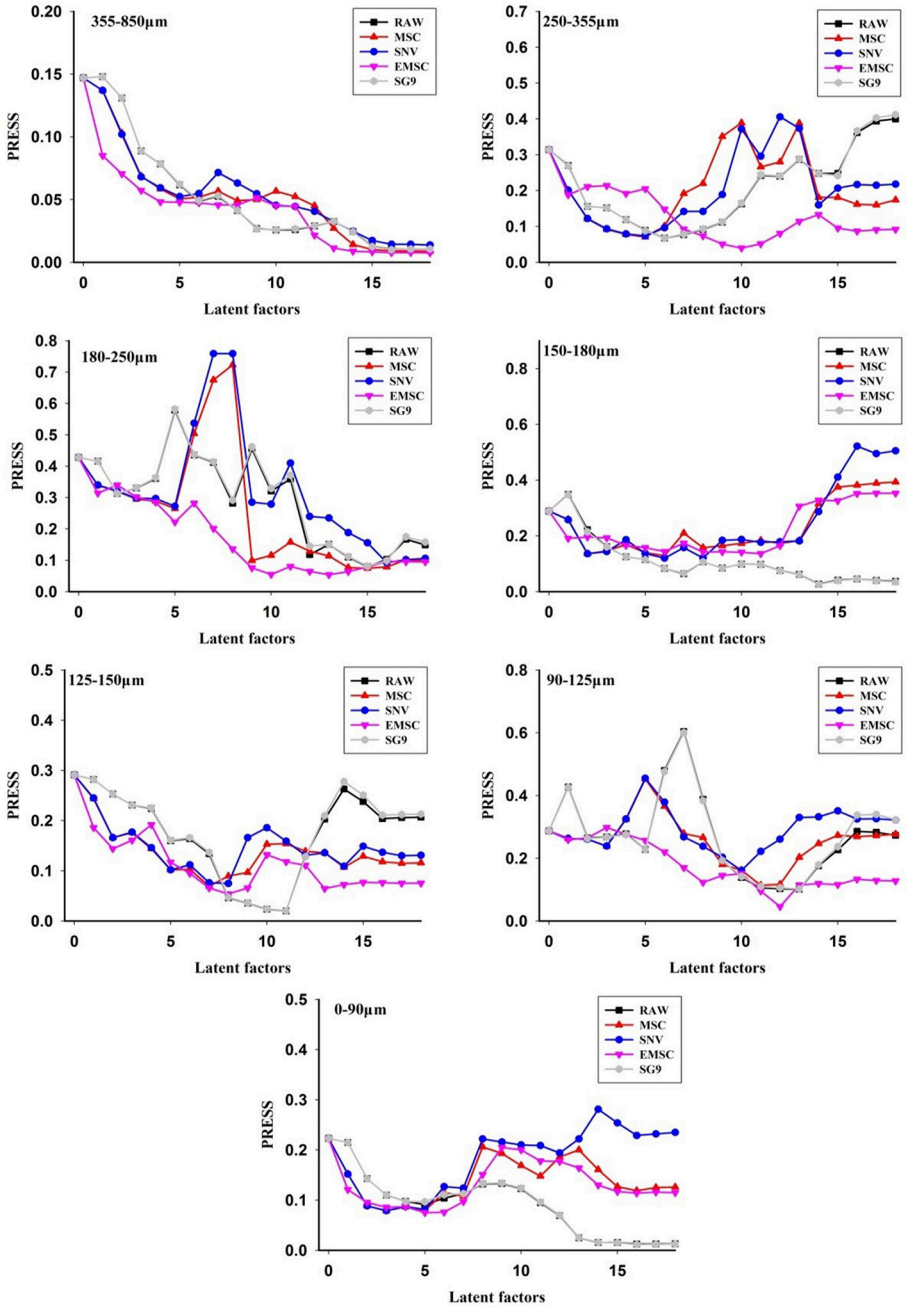
The PRESS values of different preprocessing methods for single particle size model.

Based on different preprocessing methods, the PLS model for each particle size was constructed. Figure 4 showed the relationship between the latent variables and PRESS for different preprocessing methods. In general, the lowest PRESS value means the best latent variables (Pan et al., 2015). The model was validated for prediction by internal sample set. Moreover, the model performance values for each particle size using different preprocessing methods are illustrated in Table 2. Data showed that the raw spectra were the best to construct the particle size model of 355–850 μm and <90 μm. While the best preprocessing method for the particle size model of 250–355 μm, 180–250 μm, 150–180 μm, 125–150 μm, and 90-150 μm was EMSC, SG9, SG9, SNV, and MSC, respectively.

**Table 2:** PLS model using preprocessing methods for different single particle sizes.

**Particle size (μm)**	**Preprocessing**	**Model evaluation parameters**						
		**RMSEC**	***R^2^***	**RMSECV**	***R^2^***	**RMSEP**	***R^2^***	**RPD**
355–850	RAW^#^	0.0576	0.9750	0.1642	0.8167	0.2094	0.7279	2.02^*^
	MSC	0.1451	0.8414	0.2248	0.6568	0.2187	0.7031	1.93
	SNV	0.1450	0.8418	0.2288	0.6444	0.2169	0.7082	1.95
	EMSC	0.1345	0.8638	0.2194	0.6730	0.2194	0.7014	1.93
	SG9	0.0575	0.9751	0.1635	0.8184	0.2098	0.7269	2.01
250–355	RAW	0.1497	0.9208	0.2601	0.7843	0.1884	0.8541	2.76
	MSC	0.1701	0.8978	0.2657	0.7750	0.2050	0.8272	2.54
	SNV	0.1704	0.8974	0.2715	0.7651	0.2051	0.8270	2.53
	EMSC	0.0625	0.9862	0.1996	0.8730	0.1643	0.8890	3.16^*^
	SG9	0.1498	0.9208	0.2602	0.7841	0.1885	0.8540	2.76
180–250	RAW	0.1050	0.9714	0.5666	0.2497	0.1729	0.9265	3.89
	MSC	0.0839	0.9818	0.3406	0.7289	0.2840	0.8017	2.37
	SNV	0.0678	0.9881	0.5278	0.3489	0.4332	0.5387	1.55
	EMSC	0.0800	0.9834	0.2339	0.8721	0.2198	0.8812	3.06
	SG9	0.1074	0.9701	0.5736	0.2312	0.1709	0.9281	3.93^*^
150–180	RAW	0.0304	0.9965	0.3150	0.6562	0.1699	0.9038	3.40
	MSC	0.2911	0.6746	0.3686	0.5291	0.3783	0.5232	1.53
	SNV	0.1566	0.9058	0.3459	0.5854	0.2484	0.7945	2.33
	EMSC	0.1436	0.9208	0.3801	0.4992	0.2609	0.7733	2.21
	SG9	0.0300	0.9965	0.3154	0.6553	0.1696	0.9041	3.40^*^
125–150	RAW	0.0362	0.9950	0.1537	0.9189	0.2224	0.7726	2.21
	MSC	0.1172	0.9477	0.2630	0.7623	0.1470	0.9006	3.34
	SNV	0.1082	0.9554	0.2760	0.7384	0.1029	0.9513	4.78^*^
	EMSC	0.0777	0.9770	0.2324	0.8145	0.1247	0.9285	3.94
	SG9	0.0362	0.9950	0.1553	0.9171	0.2225	0.7722	2.21
90–125	RAW	0.0644	0.9840	0.3724	0.5164	0.1722	0.7574	2.14
	MSC	0.0604	0.9859	0.4020	0.4365	0.1460	0.8257	2.52
	SNV	0.0612	0.9855	0.4016	0.4376	0.1728	0.7557	2.13
	EMSC	0.0833	0.9732	0.3505	0.5718	0.1655	0.7760	2.23
	SG9	0.0651	0.9836	0.3768	0.5049	0.1715	0.7596	2.15
<90	RAW	0.0620	0.9809	0.3505	0.4493	0.1298	0.8600	2.82^*^
	MSC	0.2352	0.7252	0.2808	0.6466	0.3437	0.0175	1.06
	SNV	0.2352	0.7253	0.2810	0.6460	0.3444	0.0133	1.06
	EMSC	0.1560	0.8791	0.2745	0.6623	0.2471	0.4920	1.48
	SG9	0.0627	0.9805	0.3523	0.4437	0.1302	0.8590	2.81

# *The original spectra without any pretreatment*.

* *The best preprocessing methods using in each different single particle size*.

**Figure 5:**
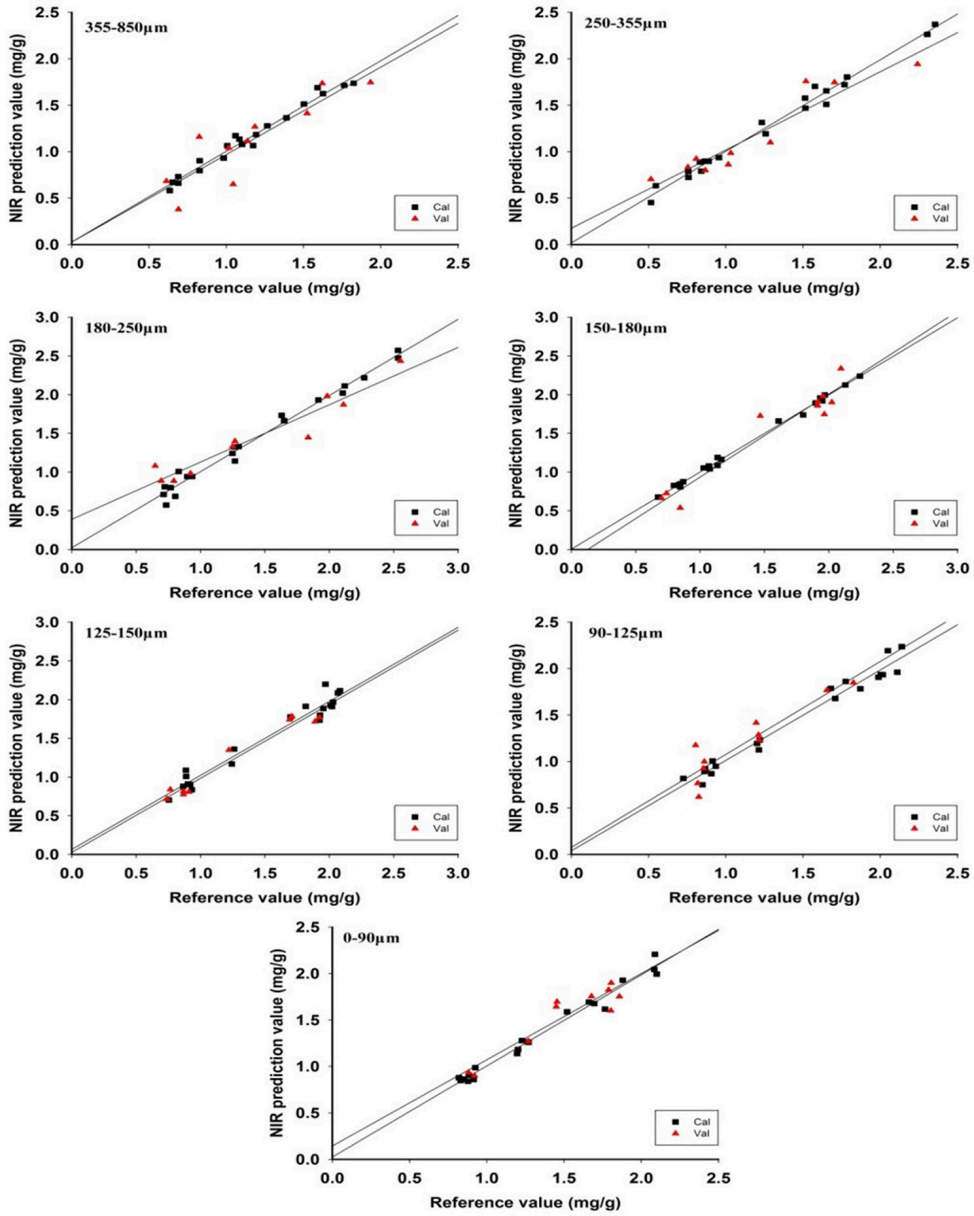
The relation map of the reference value and predicted value using each different particle size.

In addition, the model evaluation parameters, i.e., RMSEC, RMSECV, RMSEP, and RPD, for the particle size of 355–850 μm was 0.0576, 0.1642, 0.2094, and 2.02, respectively. The parameter values of other particle sizes are summarized in Table 2. The relation map between predicted value and reference value is shown in Figure 5, indicating that the best prediction result was for the particle size of 125–150 μm. Therefore, it could be known that the NIR model was influenced by different particle sizes and its quantitative characteristics was explored according to different particle sizes.

### PLS models for NIR diffuse reflectance data using *Scrophularia* radix of mix particle size

**Figure 6:**
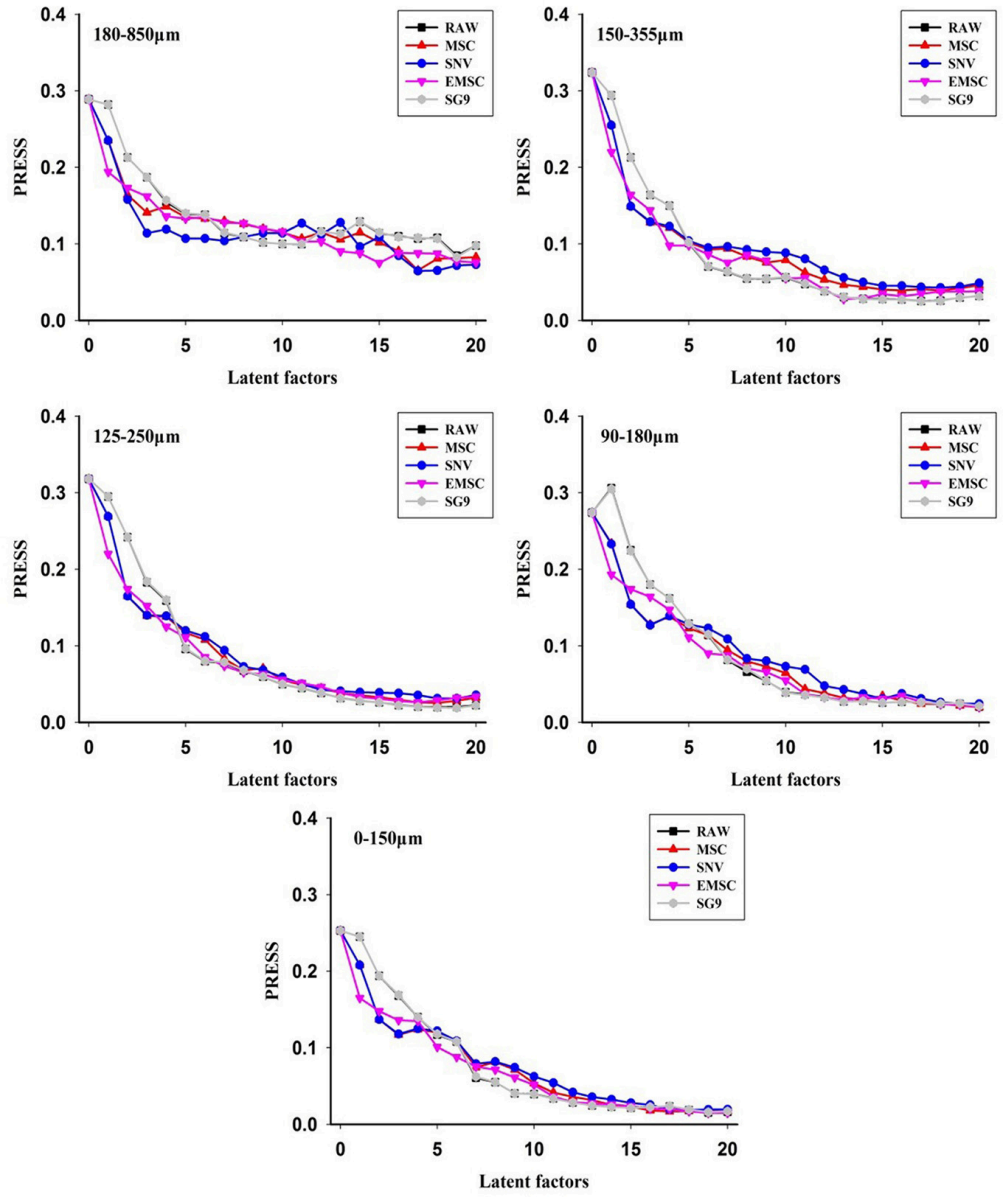
The PRESS values of different preprocessing methods for mix particle size model.

The comparison of model performance for different types of mix particle size (i.e., seven, six, five, four, and three types of particle size) manifests that the mix particle size model was best constructed for 3-type mix particle size (Table 3). Preprocessing methods were also various, such as MSC, SNV, EMSC and SG9. It can be seen from Figure 6, the optimum preprocessing method for the mixed particle size of 180–850 μm, 150–355 μm, 125–250 μm, 90–180 μm, and 0–150 μm was SG9, untreated original spectra, EMSC, EMSC, and MSC, respectively, as this model has the lowest PRESS value.

**Table 3:** Preprocessing methods for different mix particle size models (3 particle size ranges).

**Mix particle size (μm)**	**Preprocessing**	**Model evaluation parameters**						
		**RMSEC**	***R^2^***	**RMSECV**	***R^2^***	**RMSEP**	***R^2^***	**RPD**
180–850	RAW^#^	0.2492	0.7777	0.3188	0.6482	0.2699	0.7426	2.00^*^
	MSC	0.2514	0.7737	0.3392	0.6016	0.3002	0.6817	1.80
	SNV	0.2861	0.7068	0.3273	0.6291	0.3172	0.6446	1.70
	EMSC	0.2319	0.8075	0.3410	0.5973	0.2781	0.7268	1.95
	SG9	0.2494	0.7773	0.3193	0.6471	0.2702	0.7421	2.00
150–355	RAW	0.1927	0.8813	0.2338	0.8311	0.2157	0.8639	2.76
	MSC	0.2039	0.8671	0.2752	0.7659	0.2594	0.8033	2.29
	SNV	0.2654	0.7748	0.3081	0.7065	0.3117	0.7159	1.91
	EMSC	0.1467	0.9312	0.2348	0.8296	0.2053	0.8767	2.90^*^
	SG9	0.1933	0.8805	0.2345	0.8300	0.2161	0.8634	2.75
125–250	RAW	0.1592	0.9175	0.2233	0.8430	0.2592	0.7923	2.23
	MSC	0.1691	0.9069	0.2358	0.8250	0.2473	0.8109	2.34
	SNV	0.1684	0.9077	0.2428	0.8145	0.2902	0.7397	1.99
	EMSC	0.1646	0.9118	0.2358	0.8251	0.2408	0.8208	2.40^*^
	SG9	0.1597	0.9171	0.2239	0.8423	0.2595	0.7918	2.23
90–180	RAW	0.1395	0.9266	0.1983	0.8565	0.1843	0.8623	2.74
	MSC	0.1721	0.8881	0.2538	0.7649	0.1926	0.8495	2.62
	SNV	0.1805	0.8771	0.2706	0.7327	0.1978	0.8413	2.55
	EMSC	0.1572	0.9067	0.2342	0.7998	0.1632	0.8919	3.09^*^
	SG9	0.1393	0.9268	0.1974	0.8577	0.1844	0.8621	2.74
0–150	RAW	0.1585	0.8975	0.2009	0.8407	0.1744	0.8272	2.45
	MSC	0.1567	0.8998	0.2315	0.7886	0.1699	0.8359	2.51^*^
	SNV	0.1655	0.8883	0.2499	0.7536	0.1757	0.8245	2.43
	EMSC	0.1553	0.9016	0.2268	0.7970	0.1736	0.8287	2.46
	SG9	0.1588	0.8971	0.2008	0.8409	0.1746	0.8268	2.44

# *The original spectra without any pretreatment*.

* *The best preprocessing method for different mix particle size models*.

The best prediction from the mix particle size model was for 90–180 μm with RPD value >3 (Table 3). The RPD values of other mix particle size models were also about 2, meaning that the model performance of the mix particle size models was similar. This result further revealed that particle size was vital to quantitative model performance of diffuse reflectance spectra using NIR sensor. In order to make the relationship clearer, a detailed comparison of the model of the single particle size and mixed particle size was summarized.

### Comparison of the model performance for single particle size and mix particle size

It can be concluded from the comparison between the single particle size and mixed particle size models that the RPD value of the former was better than the latter. Although the prediction results were good in the prediction performance in a certain particle size range by using a single particle size model, the prediction results of single particle size model were not stable. Most of the applications of NIR diffuse reflectance spectra were for a relatively broad range of particle sizes. As a result, a mix particle size calibration model was used for prediction in subsequent studies.

Moreover, the mix particle size correction model was also used to predict the validation set for each particle size for examining which particle size samples could be more accurately predicted as well as achieving the guideline for subsequent sample preparation. The model for particle size of 90–180 μm was selected to predict the particle size of 150–180 μm, 125–150 μm, and 90–125 μm and the best preprocessing method is MSC (Table 4) and RPD values of the three prediction models are 3.81, 5.78, and 2.81 (Table 5).

**Table 4:** The prediction model for the single particle size by using the mix particle size model.

**Mix particle size(μm)**	**Preprocessing**	**Validation (150–180)**			**Validation (125–150)**			**Validation (90–125)**		
		**RMSEP**	***R^2^***	**RPD**	**RMSEP**	***R^2^***	**RPD**	**RMSEP**	***R^2^***	**RPD**
90–180	RAW^#^	0.2484	0.7945	2.33	0.1172	0.9369	4.20	0.1626	0.7839	2.27
	MSC	0.2109	0.8519	2.74	0.1723	0.8634	2.85	0.1670	0.7721	2.21
	SNV	0.2301	0.8237	2.51	0.1602	0.8820	3.07	0.1968	0.6832	1.87
	EMSC	0.1499	0.9243	3.81	0.0850	0.9668	5.78	0.1328	0.8817	2.81^*^
	SG9	0.2482	0.7949	2.33	0.1185	0.9354	4.15	0.1624	0.7844	2.27

# *The original spectra without any pretreatment*.

* *The best prediction model for the single particle size by using the mix particle size model*.

**Table 5:** Predicted results of different samples of single Scrophulariaceae Radix particle size model and calibration particle size model.

**Particle size (μm)**	**Single calibration model**			**Mix calibration model**		
	**${\text{R}}_{\text{pre}}^{2}$**	**RMSEP**	**RPD**	**${\text{R}}_{\text{pre}}^{2}$**	**RMSEP**	**RPD**
150-180	0.9041	0.1696	3.40	0.9243	0.1499	3.81
125-150	0.9513	0.1029	4.78	0.9668	0.0850	5.78^*^
90-125	0.8257	0.1460	2.52	0.8817	0.1328	2.81

* *The best predicted results*.

**Figure 7:**
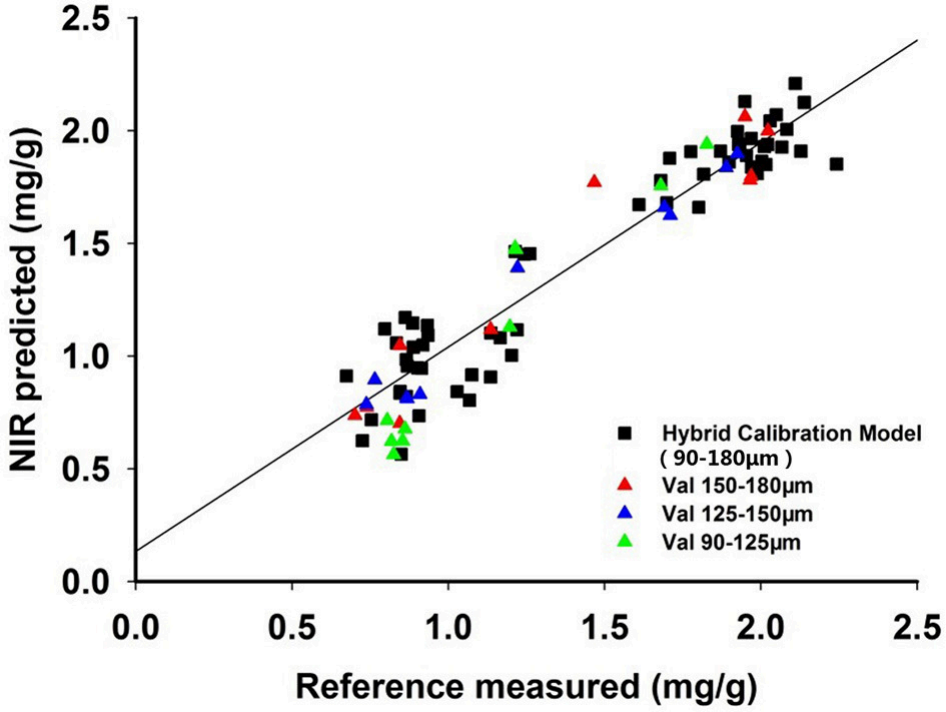
The relation map of the calibration particle size models.

On the other hand, the RPD values of the models of single particle size were 3.40, 4.78, and 2.52. Compared with the single particle size model, the RPD value of the mix particle size model was better illustrating that the prediction of the mix particle size correction model was more accurate (Table 5). The relation map between the reference and validation sets was shown in Figure 7. The correlation between reference and prediction values was good, which further demonstrated that the mix particle size model was better than the single particle size model. Why particle size was of great importance to the quantitative model of the NIR diffuse reflectance? It was performed by the Kubelka-Munk theory, which is a critical theory in the NIR diffuse reflectance.

### Discovery of the linear region of NIR diffuse reflectance spectra using the Kubelka-Munk theory

**Figure 8:**
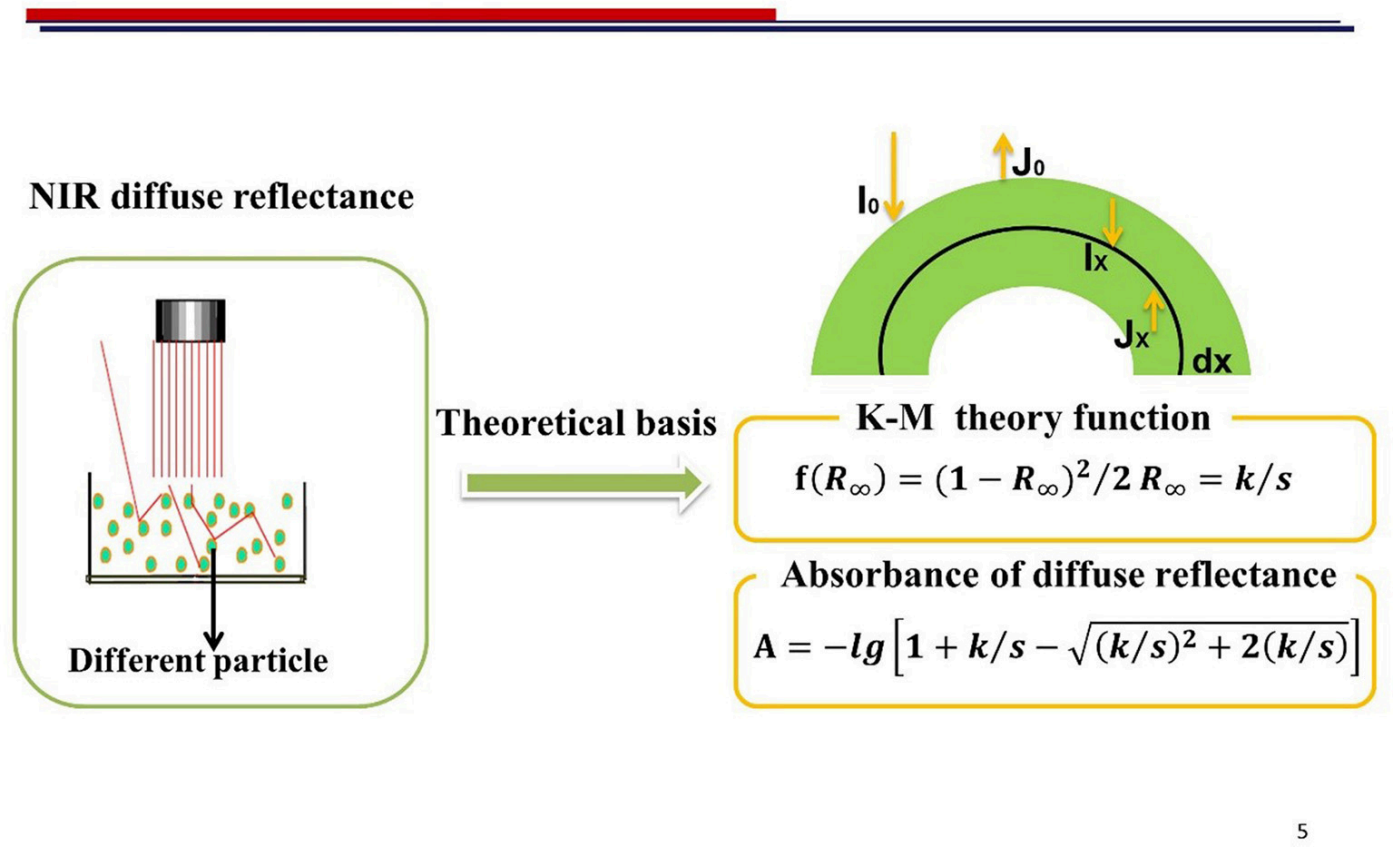
The NIR diffuse reflectance and Kubelka-Munk theory.

In practice, NIR diffuse reflectance is usually used for solid particle determination and its quantitative evidence is based on the Kubelka-Munk theory (Figure 8).

**Figure 9:**
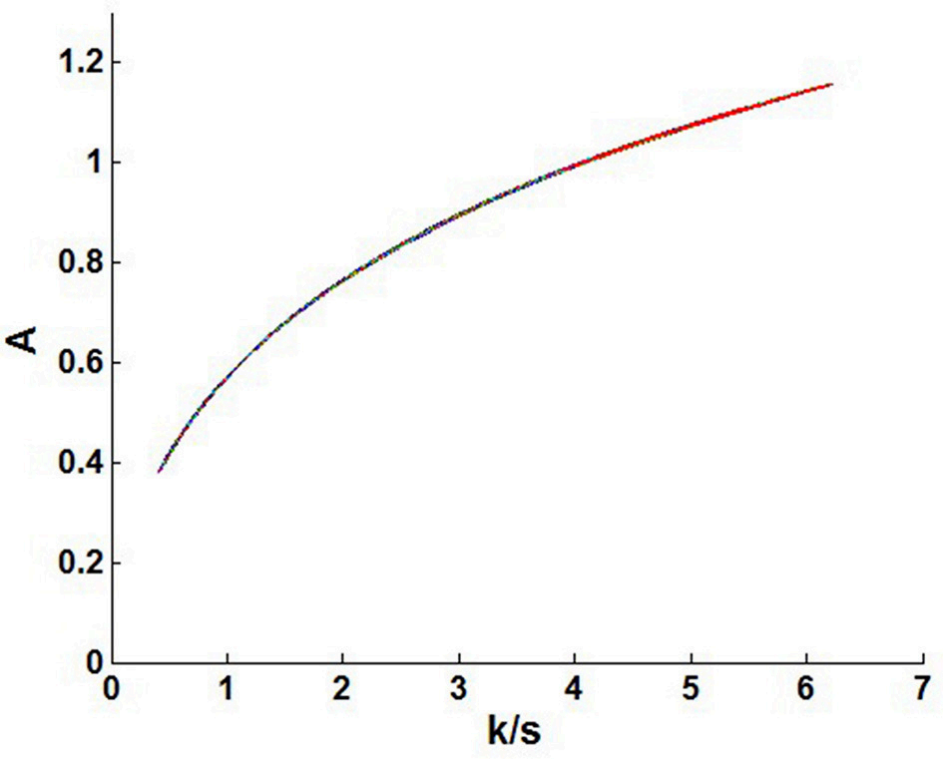
The relationship between the absorbance (A) and k/s value.

It can be learnt from the equation that the absorbance had relationship with the *k/s* value. A linear relationship was discovered between *k/s* value and *A* within a certain range. As illustrated in Figure 9, the value for *k*/s was >4 obviously indicating that a linear region existed. This results also explained and guided the modeling performance of NIR diffuse reflectance. It was found that such a linear region provides a reference for the linear modeling of diffuse reflectance spectra. It is important to note that the linear region is beneficial for establishing a NIR diffuse reflectance model. According to our data, when the scatter coefficient *s* does not change, the absorption coefficient *k* is proportional to the sample concentration. In this study, the quantitative models for single particle size and mix particle size were both constructed to minimize the limitation that the particle size of samples was only available in a certain range. The model of single particle size was better than the mix particle size owing to a small change in the scattering coefficient *s*.

## Conclusions

Particle size is of great importance to the quantitative model of the NIR diffuse reflectance. In this study, the single particle size and mix particle size models of *Radix Scrophulariae* were constructed using PLS methods. For the single particle size model, it was obvious that the best prediction model was for the particle size distribution of 125–150 μm. This particle size distribution illustrated that small particle size was beneficial to construct the quantitative model of harpagoside in *Radix Scrophulariae*.

For the mix particle size model, a better prediction was obtained for the particle size distribution of 90–180 μm indicating that the mix particle size model could explain more variation in the sample, and the accuracy and robustness of the mix particle size model would be improved. Meanwhile, the quantitative evidence of NIR diffuse reflectance of different particle sizes was based on the Kubelka-Munk theory. A linear relationship was discovered between *k*/*s* value and *A* within a certain range. Data showed that a narrow range of the scatter coefficients *s* resulted in a better model. Besides, the value for *k*/*s* was >4 clearly indicating that a linear region exited. This linear region helped explain and guide the modeling performance of NIR diffuse reflectance data. Finding such a linear region provided a methodological reference for the linear modeling of NIR diffuse reflectance spectra. Thus, further accurate assessment should be obtained in advance for a precise linear model.

Our study also showed that the quantitative analysis of CHM samples was more accurate when the scattering coefficient *s* remains unchanged or differs insignificantly at theoretical level.

## Author contributions

ZW and YQ: conceived the research; XP: performed the experiment; SD: wrote the manuscript; CD, LM, and XH: analyzed the data. All the authors prepared the manuscript and discussed the results.

### Conflict of interest statement

The authors declare that the research was conducted in the absence of any commercial or financial relationships that could be construed as a potential conflict of interest.

## Abbreviations

NIR: Near Infrared Diffuse
PAT: Process Analytical Technology
DRIFTS: Diffuse Reflectance Mid-infrared Fourier Transform Spectroscopy
CHM: Chinese Herbal Medicine
NIRS: NIR Spectroscopy
MVA: Multivariate Data Analysis
HPLC: High Performance Liquid Chromatography
RMSEC: Root Mean Square Error of Calibration
RMSECV: Root Mean Square Error of Cross-Validation
RMSEP: Root Mean Square Error of Prediction
MSC: Multiplicative Scatter Correction
SNV: Standard Normal Variate
1D: First Derivative
2D: Second Derivative
SG: Savitzky–Golay
PRESS: Predicted Residual Sum of Squares
PLS: Partial Least Squares
SCOT: Second Overtones Region
FCOT: First Combination-Overtone
RPD: Residual Predictive Deviation
API: Active Pharmaceutical Ingredient
EMSC: Extended Multiplicative Scatter Correction.
